# Anaerobic degradation of organic carbon supports uncultured microbial populations in estuarine sediments

**DOI:** 10.1186/s40168-023-01531-z

**Published:** 2023-04-20

**Authors:** Tiantian Yu, Weichao Wu, Wenyue Liang, Yinzhao Wang, Jialin Hou, Yunru Chen, Marcus Elvert, Kai-Uwe Hinrichs, Fengping Wang

**Affiliations:** 1 School of Oceanography, Shanghai Jiao Tong University, Shanghai, 200240 China; 2 State Key Laboratory of Microbial Metabolism, School of Life Sciences and Biotechnology, Shanghai Jiao Tong University, Shanghai, 200240 China; 3 Organic Geochemistry Group, MARUM-Center for Marine Environmental Sciences, University of Bremen, 28359 Bremen, Germany; 4 Faculty of Geosciences, University of Bremen, 28359 Bremen, Germany; 5 Shanghai Engineering Research Center of Hadal Science and Technology, College of Marine Science, Shanghai Ocean University, Shanghai, 201306 China

**Keywords:** Estuarine sediment, Organic carbon, Anaerobic degradation, Uncultured bacteria, Acetogenesis

## Abstract

**Background:**

A large proportion of prokaryotic microbes in marine sediments remains uncultured, hindering our understanding of their ecological functions and metabolic features. Recent environmental metagenomic studies suggested that many of these uncultured microbes contribute to the degradation of organic matter, accompanied by acetogenesis, but the supporting experimental evidence is limited.

**Results:**

Estuarine sediments were incubated with different types of organic matters under anaerobic conditions, and the increase of uncultured bacterial populations was monitored. We found that (1) lignin stimulated the increase of uncultured bacteria within the class Dehalococcoidia. Their ability to metabolize lignin was further supported by the presence of genes associated with a nearly complete degradation pathway of phenolic monomers in the Dehalococcoidia metagenome-assembled genomes (MAGs). (2) The addition of cellulose stimulated the increase of bacteria in the phylum *Ca.* Fermentibacterota and family Fibrobacterales, a high copy number of genes encoding extracellular endoglucanase or/and 1,4-beta-cellobiosidase for cellulose decomposition and multiple sugar transporters were present in their MAGs. (3) Uncultured lineages in the order Bacteroidales and the family Leptospiraceae were enriched by the addition of casein and oleic acid, respectively, a high copy number of genes encoding extracellular peptidases, and the complete β-oxidation pathway were found in those MAGs of Bacteroidales and Leptospiraceae, respectively. (4) The growth of unclassified bacteria of the order Clostridiales was found after the addition of both casein and cellulose. Their MAGs contained multiple copies of genes for extracellular peptidases and endoglucanase. Additionally, ^13^C-labeled acetate was produced in the incubations when ^13^C-labeled dissolved inorganic carbon was provided.

**Conclusions:**

Our results provide new insights into the roles of microorganisms during organic carbon degradation in anaerobic estuarine sediments and suggest that these macro and single molecular organic carbons support the persistence and increase of uncultivated bacteria. Acetogenesis is an additional important microbial process alongside organic carbon degradation.

Video Abstract

**Supplementary Information:**

The online version contains supplementary material available at 10.1186/s40168-023-01531-z.

## Introduction

Estuaries and shallow continental shelves receive large amounts of organic carbon (OC) from terrestrial and marine sources. Consequently, nearshore marine sediments play an important role globally in organic carbon burial and diagenesis and account for ∼45% of total organic carbon burial in marine sediments [1, 2]. The degradation/mineralization of OC in sediments follows the microbial respiration cascade as oxygen, nitrate, manganese, iron, sulfate, and carbon dioxide, which are subsequently used as electron acceptors with decreasing availability of Gibbs free energy [3]. In estuarine environments with high organic loading, oxygen, nitrate, manganese, iron, and sulfate are quickly exhausted within a few tens of centimeters, followed by an extensive methanogenic zone (MZ) where methanogenesis occurs predominantly [4, 5]. Microbially mediated degradation of organic carbon is a complex process during which larger macromolecules are initially broken down into monomers or oligomers that are subsequently fermented to low molecular weight intermediates such as H_2_, alcohols, lactate, acetate, propionate, and butyrate. These intermediates are eventually metabolically converted to CH_4_ and CO_2_ [6], and methanogenesis in the MZ is the terminal step of microbial mineralization of organic carbon. In the upper MZ, a large proportion of OC [7], comprising macromolecular carbohydrates, proteinaceous compounds, aromatic compounds, and humic substances, remains undegraded and buried in the MZ [8, 9]. Most studies investigating the MZ have focused on methanogenesis and metabolism of the low molecular weight intermediates [10–13], while its upstream process about the degradation of the residual higher molecules and the responsible microbes remain undetermined.

The microorganisms in marine sediments are dominated by uncultured groups such as archaea affiliated with *Ca.* Bathyarchaeota, *Ca.* Woesearchaeota, Lokiarchaeota, and Thermoplasmatales; and bacteria affiliated with Chloroflexi, Atribacteria, *Ca.* Fermentibacterota, Planctomycetes, Clostridia, and Bacteroidetes [14–24]. However, information on the metabolic properties and functions of these above-mentioned uncultured microbes was left with little understanding. Most studies are based on the predictions following the metagenome-assembled genome (MAG) analyses, which suggests that they might play important roles in OC degradation. For example, the identified genes of different encoded enzymes associated with (1) the degradation, transport, and utilization of detrital proteins in *Ca.* Bathyarchaeota and Lokiarchaeota [25–27]; (2) the degradation of aromatic compounds in Chloroflexi, Lokiarchaeota, and *Ca.* Bathyarchaeota [28–30]; (3) the fermentation of carbohydrates in *Ca.* Fermentibacterota [23]; and (4) the anaerobic degradation of hydrocarbon in Atribacteria [31]. To further illustrate the roles of the uncultivated microbes in the degradation of sedimentary OC and microbial interactions in the ecosystem, culture-dependent experimental evidence such as incubation or stable isotope probing approaches are needed.

Acetate is the key microbial metabolite in the carbon cycling of anoxic marine sediments. Besides macromolecular fermentation or hydrolysis, acetogenesis through CO_2_ reduction is considered to be an important microbial process in anoxic sediments [32–34]. Acetogenesis is the process by which acetate is synthesized de novo through CO_2_ reduction by the “Wood–Ljungdahl” (WL, reductive acetyl CoA) pathway, where various energy substrates act as electron donors, such as H_2_, CO, formate, lactate, methanol, syringate, and vanillate [34]. The capability of acetogens to metabolize different types of energy substrates coupled with CO_2_ reduction provides them ecological advantages [33]. For example, acetogenic microbes using methoxylated aromatic compounds (e.g., syringate and vanillate) can avoid competing for substrates in the environment with sulfate reducers and methanogens because they normally use formate, lactate, and methanol [34, 35]. The WL pathway is widely distributed in the metagenomes of marine sediments [25, 36] and also in various sedimentary archaeal and bacterial genomes [37, 38]. Incubation with ^13^C-labeled bicarbonate showed acetogenic growth of a bathyarchaeotal group with lignin as an energy source and CO_2_ as a carbon source for biomass accumulation and acetate production [24]. The DNA-based stable isotope probing (DNA-SIP) and gene expression data demonstrated acetogenic activity of Lokiarchaeota, whereby fermentative H_2_ production from organic substrates is coupled with the WL carbon fixation pathway [27]. The ability to degrade OC coupled to acetogenesis gives the members of *Ca.* Bathyarchaeota and Lokiarchaeota a competitive advantage to distribute globally in marine sediments. However, it remains unclear if acetogenesis is a common metabolic strategy in subsurface sediments.

Therefore, this study aims to address the following questions: (1) could the higher molecule organic compounds in the deep MZ of marine sediment be further degraded by microbes? and by which type of microbes? (2) which pathways are used by these microbes? and (3) is acetogenesis a common strategy of sedimentary microbes? We hypothesize that subsurface microbes have the capability of utilizing diverse types of organic compounds through the combination of organic carbon degradation and acetogenesis in MZ. To address these questions and test the hypothesis, we had set up a series of enrichments from estuarine sediments under anaerobic conditions that mimicked the MZ, with the addition of diverse OC substrates and ^13^C-labeled dissolved inorganic carbon. These OC substrates include the long-chain fatty acid oleic acid, the protein casein, the phenolic polymer lignin, and the polymeric carbohydrate cellulose, which represent commonly occurring organic matter in estuarine sediments [24]. In our previous study, we monitored the growth of uncultivated *Ca.* Bathyarchaeota following stimulation by the addition of lignin [24]. This study builds on the previous study by monitoring the response of various uncultured microbial groups as well as the intensity of acetogenesis in the above-mentioned substrate amendments.

## Materials and methods

### Sample collection and incubation conditions

Intertidal sediments were collected from Dayangshan island (30.592817 N, 122.083493 E) in the Hangzhou Bay of the East China Sea, and samples from 10 cm below the surface were used for incubations [24]. The samples were kept in oxygen-free gas-tight bags on ice and transported to the laboratory within 3 h, then stored at 4 °C until further processing.

The set-up of enrichment with various organic carbon substrates is described in another study [24]. Before incubation, the sediments were washed twice with NaHCO_3_-free and Na_2_SO_4_-free artificial seawater medium [39], thus diluting sulfate and dissolved organic carbon (DOC) present in the original porewater. Then, the sediment was mixed again with an anaerobic artificial seawater medium (NaHCO_3_-free and Na_2_SO_4_-free) and divided them equally. To test if inorganic C is transformed into acetate, 5 mM NaHCO_3_ containing 5% (mol/mol) ^13^C was added to one half of the samples, 5 mM NaHCO_3_ without ^13^C was added to the other half, and then the samples were dispensed into serum bottles as sediment slurries (100 mL). The ratio of liquid to sediment in slurries was about 10:1. The following five organic substrates were added to different experimental set-ups with a final concentration of 50 mg/L in slurries: oleic acid (Sinopharm, China), casein (Sinopharm, China), lignin (Sigma Aldrich, China), and cellulose (Sinopharm, China). Two replicates of each treatment were applied; the experimental setups without the addition of any organic substrate were used as controls. The slurries were incubated horizontally in the dark at 20 °C for 3.5 months (t_3.5_) without shaking. Later, a higher concentration of organic substrate solution (the final concentration was 500 mg/L for each substrate) and 0.28 mM ^13^C labeled NaHCO_3_ was added to the slurries and incubated for another 2.5 months (*t*_6_). At *t*_6_, more organic substrates (the final concentration was 500 mg/L for each substrate) were added again. Twenty milliliters of each slurry was collected after 6 (*t*_6_) and 11 (*t*_11_) months of incubation, and the samples were centrifuged at 13,800 × g for 10 min to separate the supernatant and the sediment and stored at − 80 °C for DNA/RNA isolation and acetate measurements. The sample list used for DNA/RNA isolation and acetate measurements is presented in Table S1.

### DNA extraction

Total DNA for amplifying the 16S rRNA genes was extracted from the ^12^C-NaHCO_3_ treatments (at *t*_6_ and *t*_11_) and the original sample using the PowerSoil DNA Isolation Kit (QIAGEN, China) (Table S1). The DNA for metagenomic sequencing was isolated from ^12^C-dissolved inorganic carbon (DIC) treatments at *t*_11_ and the original sample using the SDS-based DNA extraction method [36, 40] (Table S1).

### RNA extraction and reverse transcription

The total RNA was extracted from the ^12^C-DIC treatments and the original sample using the RNeasy PowerSoil Total RNA kit (QIAGEN, China). In ^12^C-DIC treatments, four samples collected at *t*_6_ and *t*_11_ of each substrate and control were mixed and used for RNA extraction (Table S1). HiScript III 1st strand cDNA Synthesis Kit (Vazyme, China) was used to perform reverse transcription. In order to exclude contaminated sequences generated during experiments, one negative control was carried out by adding sterilized water as a template from the RNA extraction. Another negative control was carried out by adding sterilized diethylpyrocarbonate (DEPC) water as the template from the reverse transcription step. The reversed cDNA from these two negative controls were also used for the amplifying and sequencing.

### Illumina sequencing and data analysis

The hypervariable V4 region of the prokaryotic 16S rRNA genes and 16S rRNA was amplified using the primer set Bac520F/Bac802R [41]. The thermal cycling program was as follows: initial denaturation at 95 °C for 4 min, 30 cycles at 95 °C for 30 s, 55 °C for 60 s, and 72 °C for 60 s, and a final extension at 72 °C for 7 min. Each reaction mixture (50 μL) contained 10 × PCR buffer, dNTPs (100 μM each), 0.25 μM of each primer, 2.5 U of DNA polymerase (Ex-Taq; TaKaRa, China), and approximately 10 ng of total DNA/cDNA. PCR products were purified using the E.Z.N.A. Gel Extraction Kit (Omega Bio-Tek, Norcross, GA, USA) following the manufacturer’s instructions. The sequence reads were obtained from the MiSeq Reagent Kit v2 (500 cycles, Illumina, San Diego CA, USA) and the Illumina MiSeq platform based on 2 × 250 bp cycles following the manufacturer’s instructions. Further analysis was performed using the QIIME 2 standard pipeline [42]. For 16S rRNA diversity analysis, the sequences in two negative controls were analyzed together with those from the experimental groups. At the species level, the groups which show high relative abundance (> 1%) in negative controls were considered as contaminated sequences and manually removed from experimental groups.

### Sequencing, assembly, binning, and annotation of the metagenome

For metagenomic analysis, paired-end sequencing was performed using a 2 × 100 bp Illumina HiSeq 2000 platform (TruSeq SBS KIT-HS V3, Illumina, at BGI-Shenzhen, China), and approximately 30G raw reads were obtained from each sample. Sickle (https://github.com/najoshi/sickle) was used to dereplicate and trim the raw shotgun sequencing metagenomic reads with the “pe” option and default setting. The dereplicated, trimmed, and paired-end DNA reads were assembled using MEGAHIT [43] with the following parameters: k-min 31, k-max 127, and step 4 [44]. The 200,145–561,880 assembled contigs, which were longer than 1 kb were obtained and used to binning into putative taxonomic groups based on abundance information using MaxBin version 2.2.4 with the run MaxBin.pl script [45]. The advantage of MAG-based metagenome analysis is that microbial genome information can be obtained without pure culture, and the disadvantage is that these genomes is incomplete and there are possible contamination sequences. The detailed estimates of genome contamination and completeness were assessed based on lineage-specific marker sets with CheckM [46]. The MAGs were classified based on the Genome Taxonomy Database (GTDB) (https://gtdb.ecogenomic.org/). All retrieved MAGs were annotated using eggnog-mapper-1.03 in the EggNOG database with *e* value 10^–10^. The MAGs were assessed for the completeness of specific pathways and functions based on the canonical pathways available in the KEGG pathway database (www.kegg.jp), and the protein family databases from Pfam 31.0. SignalP [47] were used for signal peptide predictions. The abundances of MAGs across enrichment cultures and original sample metagenomes were estimated according to the method published by Pérez Castro [48]. Metagenomic reads belonging to bacterial and archaeal 16S rRNA genes were sorted using SortMeRNA v2.1b [49] and were taxonomically classified using the SILVA SSU138 database (https://www.arb-silva.de/). Specifically, the SILVA SSU 138 database was downloaded, and a local nucleotide database for BLASTN was constructed.

### Acetate concentration and carbon isotope measurement

Before conducting the analysis, 450 µL supernatant of each type of incubated sediment slurry was acidified with 50 µL of concentrated H_3_PO_4_ (85%) to remove ^13^C-labeled dissolved inorganic carbon (DIC). The concentration and the stable carbon isotopic composition of acetate were analyzed by liquid chromatography coupled to isotope ratio mass spectrometry (LC-IRMS), following the method described by Heuer et al. [32]. Accordingly, the detection limit for quantitative analysis is 5 µM and for precise δ^13^C analysis of unlabeled samples 10 µM [32]. Since the uptake of ^13^C can be unambiguously detected in samples with acetate concentrations as low as 4 µM, we also report the δ^13^C values for samples with a concentration lower than 10 µM (Table 1). The standard deviation of replicate δ^13^C analysis of incubated samples ranged between ± 0.2 and 400‰ dependent on low or high ^13^C-label, respectively, which is consistent with previous observations by Aepfler et al. [50]. The standard deviation for concentration analysis ranged from 1 to 30 µM. Some samples were analyzed once due to sample limitation.

**Table 1:** Carbon isotopic composition and amount of acetate in the supernatant after incubation. Data were average with standard deviation (SD) after duplicate analysis; those without SDs were analyzed once; n.d., not determined; *t*_6_ and *t*_1__1_ refer to the harvest time of samples after 6 months and 11 months, respectively

Conditions	^13^C-DIC	time	δ^13^C (‰; VPDB)	Concentration (µM)	Conditions	^13^C-DIC	time	δ^13^C (‰; VPDB)	Concentration (µM)
Control	NO	*t*_6_	− 8.2	6	Casein	NO	*t*_6_	− 2.2	8.6
		*t*_6_	− 10.8	5			*t*_6_	− 4.3	4.4
	YES	*t*_6_	1740	7		YES	*t*_6_	920	79
		*t*_6_	1530 ± 400	6 ± 4			*t*_6_	730	102
	NO	*t*_11_	− 28.1	20		NO	*t*_11_	− 9.8	1303
		*t*_11_	− 21.6	11			*t*_11_	− 9.8	1189
	YES	*t*_11_	13	4		YES	*t*_11_	570	1049
		*t*_11_	n.d	n.d			*t*_11_	450	979
Cellulose	NO	*t*_6_	n.d	n.d	Lignin	NO	*t*_6_	− 14.5 ± 6.5	5 ± 2
		*t*_6_	n.d	n.d			*t*_6_	− 19.9 ± 8.2	6 ± 2
	YES	*t*_6_	990	65.2		YES	*t*_6_	530 ± 10	34 ± 1
		*t*_6_	1410	40.1			*t*_6_	1990 ± 230	5 ± 2
	NO	*t*_11_	− 15.3	27.0		NO	*t*_11_	− 15.9	26
		*t*_11_	− 18.7	289			*t*_11_	− 17.2	44
	YES	*t*_11_	560	219		YES	*t*_11_	510 ± 80	89 ± 10
		*t*_11_	540	10.5			*t*_11_	1040 ± 40	36 ± 5
oleic acid	NO	*t*_6_	18.0 ± 0.4	280 ± 30					
		*t*_6_	19.0	131					
	YES	*t*_6_	1500	193					
		*t*_6_	1590	29					
	NO	*t*_11_	− 4.0	335					
		*t*_11_	− 9.2	414					
	YES	*t*_11_	910	301					
		*t*_11_	390	1137					

## Results

Our previous study [24] focused only on the growth of the members of *Ca.* Bathyarchaeota stimulated by lignin. This study elucidated the growth responses of different types of sedimentary microbes to amendments of structurally diverse organic compounds. Four substrates, including oleic acid, casein, lignin, and cellulose served as model compounds for the common sedimentary constituents, i.e., long-chain fatty acids, proteins, aromatic compounds, and polymeric carbohydrates, respectively [24]. Casein and cellulose stimulated the growth of microbes, while microbial growth was not stimulated by oleic acid and lignin (Fig. S1). This was probably because the organic matter of proteins and carbohydrates facilitated the division and proliferation of microbial cells.

### The shift in archaeal and bacterial communities across treatments with OC

**Fig. 1:**
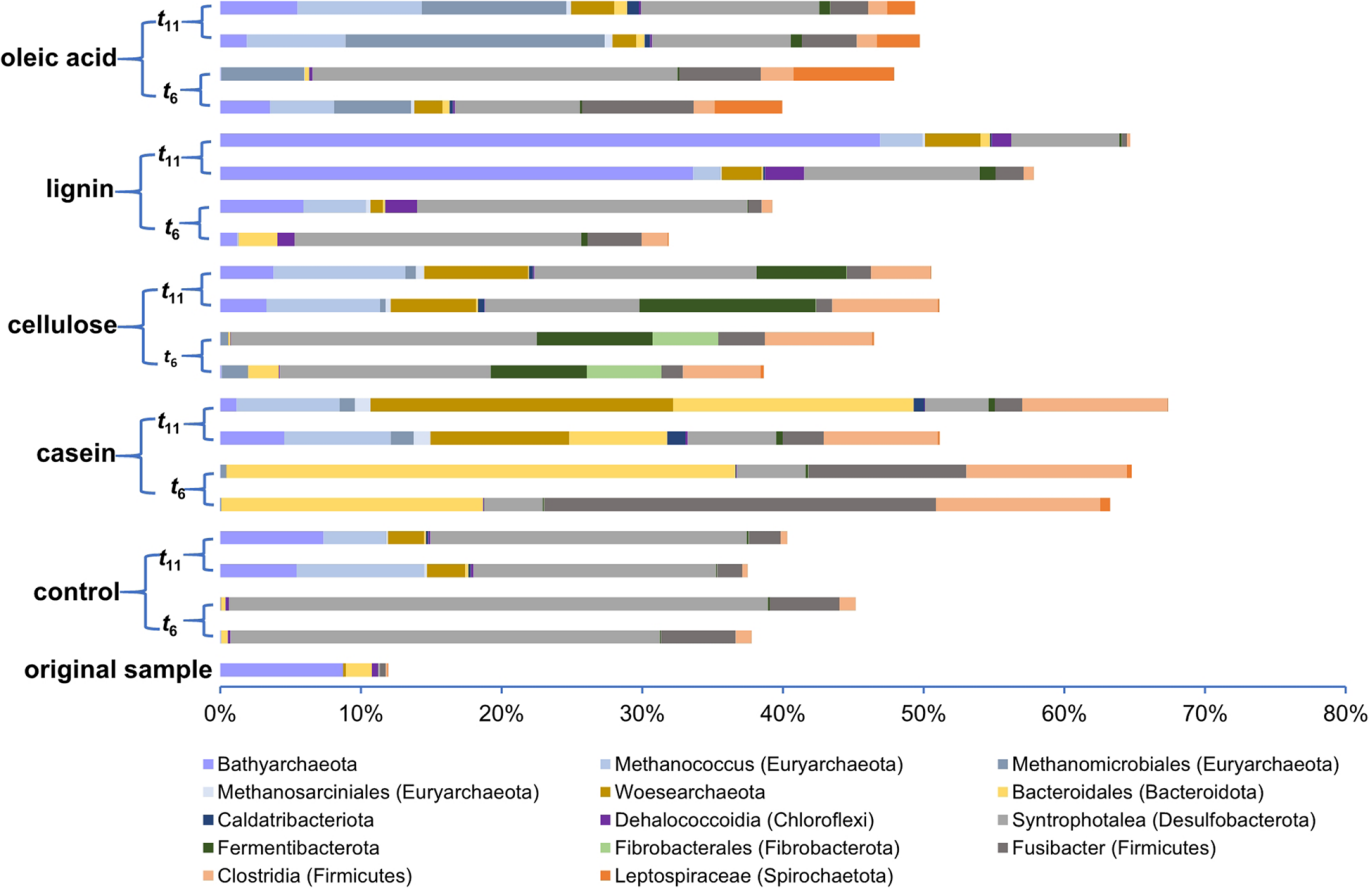
The relative abundance of microbial groups showing a response to the addition of different OMs based on analysis of 16S rRNA gene amplicons. Shown is the increase in the relative abundance of *Methanococcus*, *Methanocalculus*, Methanosarcinales, *Ca.* Bathyarchaeota, *Ca.* Woesearchaeota, *Ca.* Fermentibacterota, Fibrobacterales, Bacteroidales, *Fusibacter*, Clostridiales, *Syntrophotalea*, Leptospiraceae, and Dehalococcoidia. *t*_6_ and *t*_11_ refer to the harvest time of samples after 6 months and 11 months, respectively

The changes in the microbial communities were determined by high-throughput prokaryotic 16S rRNA gene amplicons, and the growth responses of some microbial groups to these amended OC were monitored. The compositions of the prokaryotic community at the phylum level in the original sediment, and after 6 and 11 months of incubation (*t*_6_ and *t*_11_), are shown in Fig. S2. Our study highlights the groups that showed growth throughout the enrichment in Fig. 1. For archaea, the relative abundance of three types of methanogens, including the members in the genus *Methanococcus*, the genus *Methanocalculus*, and the order Methanosarcinales, increased in all treatments (Fig. 1). Besides the abundance of the uncultured phylum of *Ca.* Bathyarchaeota influenced by lignin [24], the relative abundance of the uncultured phylum of *Ca.* Woesearchaeota increased substantially after treatment with casein and cellulose. The members of the phylum *Ca.* Woesearchaeota have been suggested to have a symbiotic lifestyle as the size of their genomes is small and their central metabolic pathways are absent [51].

Regarding bacteria, the relative abundances of the groups in the genus *Fusibacter* (Phylum Firmicutes) and the genus *Syntrophotalea* (Phylum Desulfobacterota) increased in all amended and unamended treatments (Fig. 1), suggesting that they might play a less prominent role in the initial degradation steps of the added organic substrates. We also found that some uncultured bacterial groups grew in response to specific substrates. The relative abundances of bacteria in the uncultured phylum of *Ca.* Fermentibacterota and unclassified groups in the family Fibrobacterales (Fibrobacterota) increased solely after cellulose treatment. Lignin treatment specifically increased the relative abundance of unclassified lineages in the class Dehalococcoidia (Chloroflexi). Casein and cellulose both stimulated the growth of the unclassified groups in the order Clostridiales (Firmicutes). Specific enrichments were also found in the unclassified groups of order Bacteroidales (Bacteroidota) and the family Leptospiraceae (Spirochaetota), which increased after treatment with casein and oleic acid, respectively (Fig. 1). Besides the relative abundances, the cell numbers of the abovementioned lineages of uncultured bacterial groups increased by several-fold to three orders of magnitude, calculated from the total prokaryotic 16S rRNA gene copy numbers and their relative abundance in the microbial community (Fig. S1).

Moreover, to verify the activity of these specific groups in the corresponding cultures, the transcripts of 16S rRNA genes were also detected. The results showed that the relative abundances of 16S rRNA in these incubated samples were similar to those of the 16S rRNA gene (Fig. S3). Cell transcription was detected for all the abovementioned growing uncultured bacteria, with particularly high levels of activity observed for the order Bacteroidales, family Fibrobacterota, and Leptospiraceae after treatment with casein, cellulose, and oleic acid, respectively. Metagenomic reads belonging to bacterial and archaeal 16S rRNA genes were also extracted for analysis of microbial composition (Fig. S4), and it showed a similar trend with these based on the 16S rRNA gene and 16S rRNA amplicon.

### Change in abundance of genes related to OC degradation

The genes related to OC degradation were retrieved from the metagenomic data, and their abundances were quantitatively compared (Fig. S5). Genes related to the β-oxidation pathway increased after the addition of oleic acid, including those encoding long-chain fatty acid acyl-CoA synthetase, acyl-CoA dehydrogenase, enoyl-CoA hydratase, and acetyl-CoA acyltransferase. The abundances of genes encoding peptidase and protease were much higher in the amendment with casein compared to those in other amendments. Genes encoding catalase-peroxidase and benzoyl-CoA reductase (*bcr*) for lignin and aromatic compound degradation were most abundant in the amendment with lignin. Likewise, genes encoding endoglucanase and cellobiose phosphorylase, responsible for cellulose degradation, were enriched by adding cellulose. The change in abundance of genes related to different OC amendments suggests a clear response of relevant functional microbes towards different OC inputs.

### OC degradation pathways in the MAGs of the specific enriched microbes

Metagenomic data were also used for assembly and binning, the obtained metagenome-assembled genomes (MAGs) were classified based on the Genome Taxonomy Database (GTDB). MAGs of these specific enriched bacteria described above, i.e., Dehalococcoidia, *Ca.* Fermentibacterota, Fibrobacterales, Bacteroidales, Leptospiraceae, and Clostridiales were selected (Table S2) and further analyzed. MAGs of Bacteroidales, Dehalococcoidia, and Leptospiraceae showed higher abundance in casein, lignin, and oleic acid treatment, respectively; MAGs of *Ca.* Fermentibacterota and Fibrobacterales showed higher abundance in the cellulose treatment; MAGs of Clostridiales showed higher abundance in the casein and cellulose treatments (Table S2). The sequences of the 16S rRNA gene were extracted from most of these MAGs which showed low similarities to those from best-matching cultivated strains (between 83.3 and 92.6%) (Table S2), indicating further that these enriched bacterial groups were yet uncultivated.

#### Dehalococcoidia

**Fig. 2:**
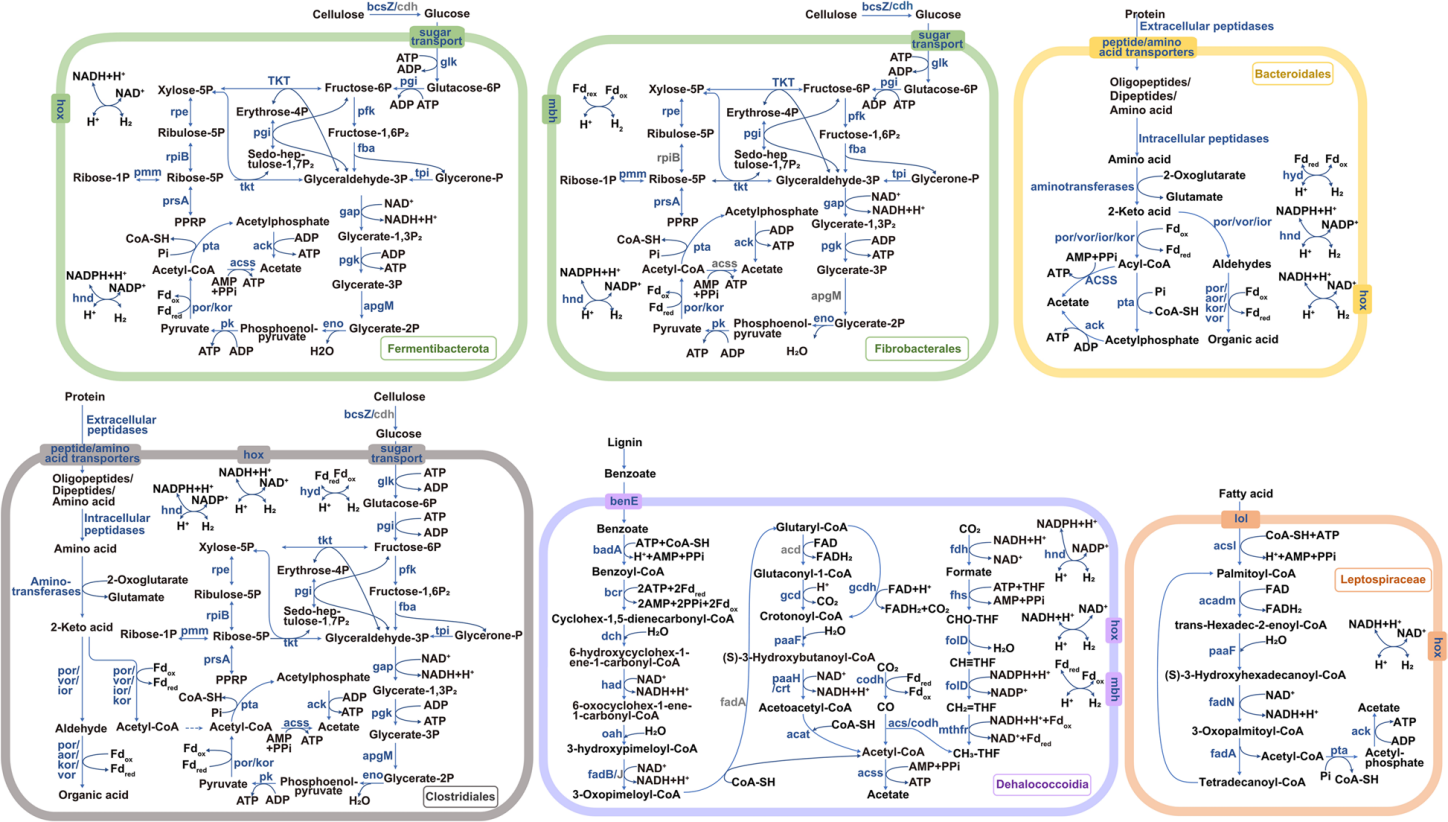
Metabolic map of uncultured bacteria illustrating the degradation pathways of added substrates to acetate and H_2_. Genes detected in our MAGs are shown in blue, and genes are not detected in our MAGs but are detected in the reference shown in gray. Enzymes are abbreviated with letters, and the full list as well as further metabolic pathways are provided in Supporting Information Table Tables S3, S4, S5, S6, S7 and S8

Besides the enrichment of *Ca.* Bathyarchaeota [24], we found the growth response of Dehalococcoidia in lignin-amended samples (Fig. 1). Seven MAGs of Dehalococcoidia were constructed (Table S2). The highest abundance of Dehalococcoidia MAG contained a nearly complete benzoate degradation pathway for the anaerobic oxidation of benzoate to acetyl coenzyme A (Fig. 2 and Table S3).

#### *Ca. *Fermentibacterota and Fibrobacterales

The abundance of uncultured bacteria in the phylum *Ca.* Fermentibacterota (formerly candidate division Hyd24-12) and family Fibrobacterales increased specifically in the cellulose-treated sample (Fig. 1). One MAG of *Ca.* Fermentibacterota and two MAGs of Fibrobacterales were constructed (Table S2). The genes coding for extracellular endoglucanase or/and 1,4-beta-cellobiosidase (*cbh*), which facilitates cellulose decomposition, were present in these MAGs with high copy numbers (13–36) (Tables S4 and S5). Moreover, the genes involved in sugar transport, including the transporters of polysaccharides, cellobiose, multiple monosaccharides, lipopolysaccharides, and maltooligosaccharides were present. The genes associated with the complete or nearly complete glycolytic and pentose phosphate pathways were also present in these MAGs (Fig. 2).

#### Bacteroidales

The growth of the members of Bacteroidales was specifically detected in the casein amendments (Fig. 1). Seven MAGs of Bacteroidales were obtained (Table S2), and all of them contained genes coding for oligopeptides, branched-chain amino acids, and proline transporters and high copy number of genes encoding extracellular (M23, M28, C69, S9, S41 etc.) and intracellular peptidases (M22, M23, M24, M29, M56, S46, S51 etc.) (Table S6). The genes coding for the intracellular breakdown of amino acids were also present, including ferredoxin-reducing oxidoreductases specific for aldehydes (*aor*), pyruvate/2-ketoisovalerate (*por*), indolepyruvate (*ior*), oxoisovalerate (*vor*), and 2-oxoglutarate (*kor*) (Fig. 2).

#### Leptospiraceae

The members of uncultured bacteria in the family Leptospiraceae were enriched specifically after the addition of oleic acid (Fig. 1). One MAG of Leptospiraceae was constructed, and it had the genetic capability to utilize fatty acids (Fig. 2 and Table S2), i.e., it possessed genes coding for complete β-oxidation and a lipoprotein transporter (*lol*) (Fig. 2 and Table S7).

#### Clostridiales

The growth of uncultured groups in the order Clostridiales was detected in both casein and cellulose treatments (Fig. 1). Four MAGs of Clostridiales were constructed (Table S2). For protein and amino acid degradation, a high copy number of genes for extracellular (S9, S11, S13, S33, S41, and M23) and intracellular (M20, M24, M28, M29, M56, and S58) peptidases and genes coding for ferredoxin-reducing oxidoreductases (including *aor*, *por*, *vor*, and *kor*) were found in these MAGs of Clostridiales (Table S8). For cellulose and glucose degradation, genes coding for endoglucanase, *cbh*, and those associated with the complete or nearly complete glycolytic and pentose phosphate pathway were identified (Fig. 2 and Table S8). Additionally, the transporter genes of the branched-chain amino acid proline, lipoproteins, oligopeptides, glucose, polysaccharides, and maltose were also identified.

### Acetate production by the fermentation of OC

In methanogenic sediments, it is well known that acetate can be produced by the fermentation of organic matter. Here, the production of acetate was observed in all OC treatments (Table 1). The concentrations of acetate in lignin and cellulose treatments were generally lower than that in the oleic acid and casein treatments (*p* = 0.0002 < 0.05) (Table 1), which was probably because lignin and cellulose are less accessible to microbes. Because of the addition of OC at *t*_6_, acetate concentrations in most OC treatments increased from *t*_6_ to *t*_11_ (Mann–Whitney test with one-tail, the same as below; *p* < 0.05). Specifically, the addition of casein stimulated acetate production 23-fold from *t*_6_ to *t*_11_ (*p* = 0.014 < 0.05). The acetate production was also detected in the controls (without OC), although their concentrations were substantially lower than in treatments with OC additions and did not differ between the time points (*t*_6_ to *t*_11_) (*p* > 0.05). The small amount of acetate present in the controls is probably due to fermentation of the remaining OC in the original sediments, and since no extra OC was added at *t*_6_, no increase was observed between *t*_6_ and *t*_11_.

### Acetate production by acetogenesis

A substantial quantity of acetate (up to ~ 1100 µM) with positive δ^13^C values (up to ~ 2000‰) was produced in all incubation lines with OC addition (Table 1). The incorporation of the ^13^C-label from ^13^C-DIC into acetate supports our assumption that acetogenesis, involving CO_2_ reduction via the WL pathway, is a secondary process that is interrelated with the degradation of OC. The lowered δ^13^C-acetate values at *t*_11_ compared to *t*_6_ (*P* = 0.01 < 0.05) go along with increased acetate concentrations, which is likely due to an increase in the generation of non-labeled DIC through the degradation of the supplied organic substrates. The control samples without OC addition also showed high δ^13^C-acetate values, suggesting that acetogenesis is common in estuarine sediments.

## Discussion

### Niches of enriched microbes involved in OC degradation in MZ

A substantial fraction of organic carbon is buried in nearshore sediments where the microbial transformation of organic carbon occurs as a key process influencing carbon flow and ultimately atmospheric oxygen and carbon dioxide concentrations [1]. In nearshore sediments with high organic matter loading, electron acceptors such as oxygen, nitrate, manganese, iron, and sulfate are quickly exhausted within the top a few millimeters to centimeters, leading to an extensive methanogenic zone (MZ) where a large proportion of organic carbon persists [4, 5]. Methanogenesis in MZ mediates 28.6% of global subseafloor organic matter degradation [52], while methanogens can only use low molecular weight intermediates as substrates [10–13], the transformation of residual higher molecules into low molecular weight intermediates, and the responsible microbes remain undetermined.

The dry weight of vascular plants comprises a large proportion (20–50%) of the organic carbon deposited into nearshore sediments [53, 54]. Cellulose is the most abundant of vascular plants derived organic matter followed by hemicellulose and lignin [55]. Here, the uncultured bacteria within the class Dehalococcoidia were stimulated in lignin-amended samples (Fig. 1 and S1). Although the known genes related to lignin polymer depolymerization were absent in Dehalococcoidia metagenome-assembled genomes (MAGs) [56], genes associated with a nearly complete degradation pathway of phenolic monomers could be found (Fig. 2 and Table S3). The catalytic processes of all the reported lignin polymer depolymerization enzymes require oxygen (aerobic environment) [56], while the mechanisms involved in the anaerobic depolymerization of lignin are unclear [57]. Therefore, there might be new and unannotated genes in the genomes of Dehalococcoidia for lignin polymer depolymerization in anaerobic environments. Another possibility could be the synergistic utilization of lignin, where depolymerization of lignin polymer was conducted by other types of microbes, and the members of Dehalococcoidia participated in the downstream degradation of the resulting lignin/phenolic monomers. The class Dehalococcoidia, belonging to phylum Chloroflexi, is widely distributed in different types of environments and is usually enriched in marine subsurface sediments [30, 58]. However, only a few strains (4 species) in this group have been cultivated, leaving its metabolic properties ambiguous. In total, 31 MAGs of Dehalococcoidia were collected from the NCBI prokaryote genome database (https://www.ncbi.nlm.nih.gov/assembly/) and analyzed along with the MAGs obtained in this study (Table S3). Contrary to the MAGs constructed from groundwater, seawater, or wastewater, etc., most MAGs derived from marine sediments contained the benzoyl-CoA reductase (*bcr*) genes, encoding the key enzyme involved in the anaerobic degradation of aromatic compounds. Thus, the marine sediment-derived Dehalococcoidia probably play an important role in the degradation of aromatic compounds including lignin derivatives.

The addition of cellulose stimulated the increase of bacteria in the phylum *Ca.* Fermentibacterota, family Fibrobacterales, and order Clostridiales (Fig. 1 and Fig S1); a high copy number of genes encoding extracellular endoglucanase or/and 1,4-beta-cellobiosidase for cellulose decomposition and multiple sugar transporters were present in their MAGs (Fig. 2, Tables S4, S5 and S8). The members of the phylum *Ca.* Fermentibacterota are typically uncultured bacteria that are globally distributed, and they are usually found in anoxic, organic, and/or methane-rich sedimentary settings, including marine sediment [23, 59–61]. Here, all MAGs of *Ca.* Fermentibacterota (a total of 18) were downloaded from the database (Table S4); these MAGs come from different environments, including marine microbial mats, marine sediments, anaerobic digestion of organic wastes, the mouth of dolphin, soil, and wastewater. All MAGs in *Ca.* Fermentibacterota contained the endoglucanase gene and the genes associated with the complete or nearly complete glycolytic and pentose phosphate pathways. Thus, the fermentation of cellulose might be their common strategy to survive in different anoxic environments, including anoxic estuarine sediments. The members of the family Fibrobacterales were the major degraders of cellulose in the herbivore gut; historically, they were thought to only occupy mammalian intestinal tracts [62]. However, 16S rRNA genes within Fibrobacterales were also detected in landfill sites and freshwater sediments, suggesting a potential role for this family in cellulose degradation beyond the herbivore gut [62]. Here, the carrying genes coding for cellulose degradation in our MAGs of the family Fibrobacterales further confirmed this assumption and revealed the possibility of their function in estuarine sediments (Fig. 2 and Table S5).

Fatty acids are ubiquitous components in marine sediments, and oleic acid is one major fatty acid in terrestrial plants and marine phytoplankton [63]. The members of the family Leptospiraceae were enriched by the addition of oleic acid (Fig. 1 and Fig S1), and the complete β-oxidation pathway was found in the MAG of Leptospiraceae (Fig. 2 and Table S7). Moreover, 12 available MAGs or genomes of the family Leptospiraceae were retrieved from the database; most of them were extracted from marine environments (Table S7). All these marine-derived genomes contained genes for complete β-oxidation suggesting that fatty acid degradation was their common ecological role and survival strategy. Proteins typically constitute 10% of the organic matter found in marine sediments [64]. The growth of unclassified bacteria of the order Bacteroidales and order Clostridiales was found after the addition of proteins (casein) (Fig. 1 and Fig S1), and their MAGs contained multiple copies of genes for extracellular peptidases (Fig. 2, Table S6 and S8), which implies their role in biomineralization of proteins.

### Acetogenesis are important contributors of acetate in marine sediments

A substantial quantity of acetate with positive δ^13^C values was produced in all incubation lines with/without OC addition (Table 1), suggesting that acetogenesis is key biogeochemical processes and important sources of acetate in estuarine sediments. Acetate usually accumulates in anaerobic marine sediments [13, 65–69], while it is widely believed that its major source is the fermentation of OC. Only a few studies have mentioned that acetate accumulates in marine sediments due to the reduction of CO_2_ by acetogens. Heuer et al. measured the δ^13^C values of acetate in methane-rich sediments at the northern Cascadia Margin and showed that acetogenic CO_2_-reduction/fixation can coexist with methanogenic CO_2_-reduction [32]. Moreover, Lever et al. proposed that besides fermentation, and CO_2_ reduction via the WL pathway might be a source of acetate in marine sediments [33, 34]. This hypothesis was supported by the fact that the WL pathway, as one of the most important pathways for carbon fixation, is widely distributed in marine sediments [36]. It is also widespread within the archaea and bacteria [38], including cosmopolitan sedimentary microbes, such as methanogenic archaea, *Ca.* Hadesarchaea, *Ca.* Theionarchaea, *Ca.* Altiarchaeales, *Ca.* Thorarchaeota, *Ca.* Bathyarchaeota, and Lokiarchaeota among archaea; and Planctomycetes, Proteobacteria, Acidobacteria, Desulfobacterota, Firmicutes, and Chloroflexi among bacteria [37]. Moreover, most microbes carrying the WL pathway also have the genes of degrading a wide range of OC, and their OC degradation metabolism might be coupled with the WL carbon fixation pathway. Under methanogenic condition, CO_2_ can be used as electron acceptor and CO_2_ fixation can consume the cellular reducing power produced by OC metabolism.

The occurrence of acetogenesis in marine sediments via the WL pathway is supported by our metagenomic data. For example, the addition of OC resulted in an increase in the relative abundance of genes encoding the carbon monoxide dehydrogenase/acetyl-CoA synthase complex (*CODH/ACS*) (subunit alpha and epsilon), the key enzyme in the WL pathway (Table S9). Besides *Ca.* Bathyarchaeota [24], the complete WL pathway and the *acss* gene/*pta-ack* pathway were also found in the MAGs of the family Desulfatiglandales (Desulfobacterota) and Dehalococcoidia (Tables S3 and S10). For the uncultured groups of Dehalococcoidia and *Ca.* Bathyarchaeota, the complete WL pathway was also frequently detected in the MAGs found in other studies [30, 36, 70]. The acetogenic growth of *Ca.* Bathyarchaeota with lignin as the electron donor for CO_2_-reduction with acetate production was shown earlier [24], and the members of Dehalococcoidia probably have an acetogenic metabolism similar to that of *Ca.* Bathyarchaeota. The members of phylum Desulfobacterota are globally distributed with numerous cultured representatives, and they have historically been classified in the class Deltaproteobacteria. Cultured strains of Desulfobacterota show a preference for anoxic conditions, and many members utilize sulfate, sulfite, thiosulfate, and elemental sulfur as the electron acceptor, with different types of OC as the electron donor [71, 72]. The WL pathway is frequently detected in the genomes of the cultured and uncultured members of Desulfobacterota [73–75]. However, in contrast to their role in sulfur reduction, their acetogenic metabolism has received little attention and has rarely been tested. The capacity to perform acetogenesis was demonstrated only in two sulfate-reducing bacteria, including *Desulfotignum phosphitoxidans* and *Dethiosulfatarculus sandiegensis*, grown in the absence of sulfate [76, 77]. In this study, the three MAGs of the family Desulfatiglandales (Desulfobacterota) carrying the genes for the complete WL pathway showed higher abundance after OC addition (Table S2). Based on our findings described above, along with the evidence provided by the carbon isotope measurements of acetate, we argue that their WL pathway might be used to fix inorganic carbon for acetate production. Taken together, our study indicates that acetogenesis through the WL pathway is an important biogeochemical process in estuarine methanogenic sediments, which has been overlooked in previous studies.

### Implications of organic matter biomineralization for marine carbon cycling

**Fig. 3:**
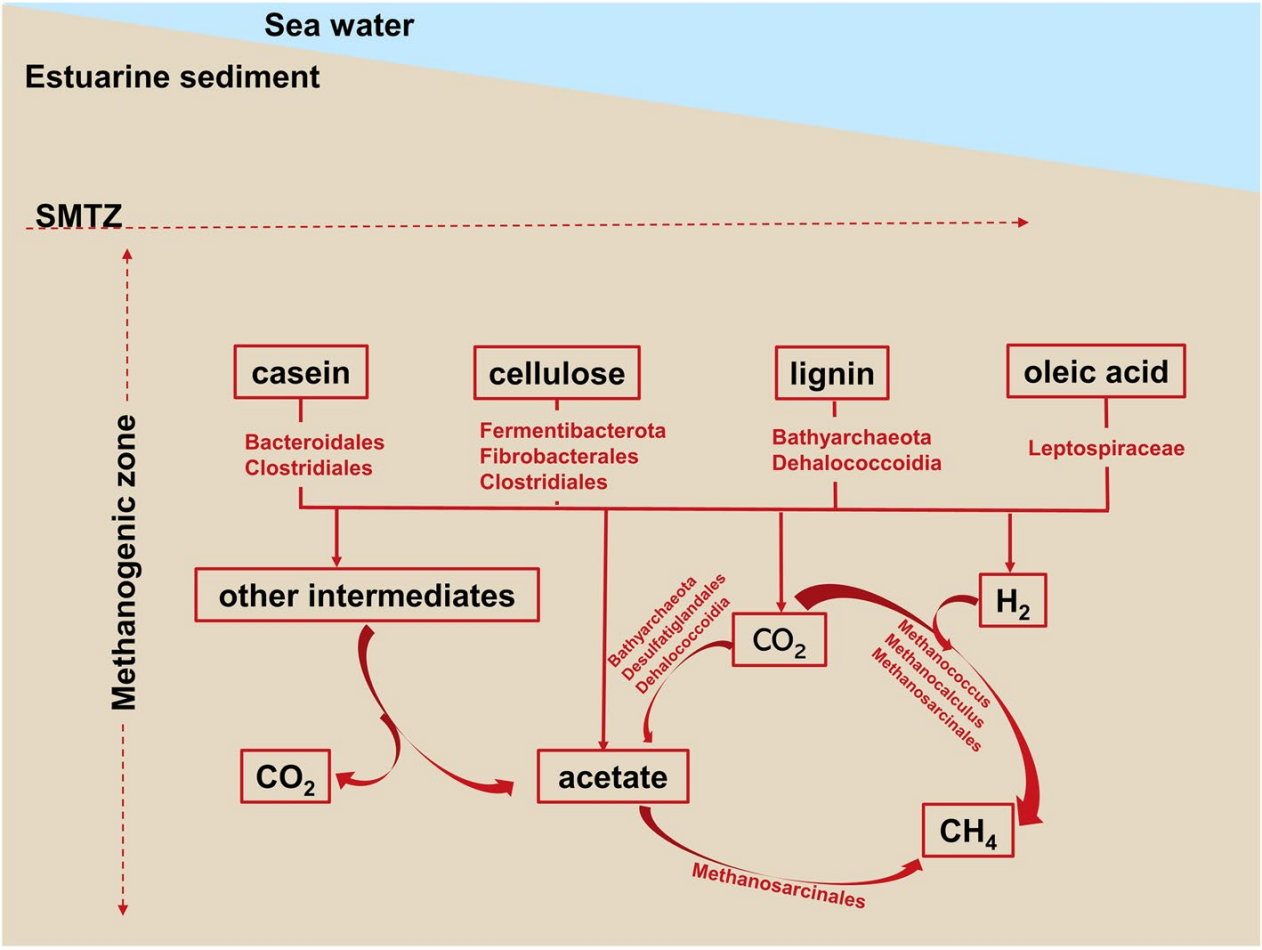
Carbon flow from the added substrates to CH_4_, CO_2_, and acetate and the identified microbial groups involved in the cycling of these compounds in estuarine methanogenic sediments

Although the concentrations of hydrogen and methane were not detected in this study, their production could be inferred from the growth of methanogens in the genus *Methanococcus*, genus *Methanocalculus*, and order Methanosarcinales. Methanogenesis is the terminal step of OC degradation; not surprisingly, the growth of methanogens in the different OC amending enrichments was found. The members in the genus *Methanocalculus* and *Methanococcus* are hydrogenotrophic methanogens, and the members in order Methanosarcinales are metabolically versatile methanogens, which can metabolize a wide range of substrates for methane production, e.g., H_2_/CO_2_, acetate, methanol, and methylamines [78–81]. Nearly complete hydrogenotrophic pathways were found in the MAGs of these three groups (Table S11). Hydrogen also was a ubiquitous intermediate of OM fermentation in marine sediments, and most MAGs of OM degraders possessed hydrogenase genes, such as NAD-reducing hydrogenase *(hox), NADP-reducing hydrogenase (hnd)*, and coenzyme F420 hydrogenase subunit beta* (frhB)* genes (Fig. 2, Tables S3, S4, S5, S6, S7 and S8), which implies their role in the production of hydrogen for methanogens (Fig. 3). Two MAGs classified into the order Methanosarcinales also contain the acetoclastic methanogenesis pathway (Table S11); therefore, they likely also consumed acetate for methanogenesis in these treatments (Fig. 3).

## Conclusions

We conducted long-term incubations (up to 11 months) of estuarine sediments with different types of organic carbon substrates, ranging from macro to single molecules, to study the microbial processes involved to understand the associated carbon flow (Fig. 3). Based on the analysis of microbial composition (data came from 16S rRNA gene and 16S rRNA amplicons) and metabolic pathway (data came from metagenome), various uncultured bacteria showed a potential response to the degradation of the supplied carbon substrates. Furthermore, the formation of ^13^C-labeled acetate from ^13^C-DIC indicated that besides the fermentation of carbon substrates, microbial acetogenesis by CO_2_ reduction is an important process in the estuarine sediments. For further examination of the identified organic carbon degraders, future studies should combine the metatranscriptomic analysis with the addition of isotopically labeled organic substrates for DNA/RNA-based stable isotope probing (DNA/RNA-SIP) analysis.

## Supplementary Information

**Additional file 1: Table S1.** The list of samples used for DNA/RNA isolation and acetate measurements. **Table S2.** The overview of MAGs that were analyzed in this study. **Table S3.** The list of genes that are associated with benzoate degradation to acetate and H_2_ production in the MAGs of Dehalococcoidia. **Table S4.** The list of genes that are associated with the cellulose degradation to acetate and H_2_ production in the MAGs of* Ca. *Fermentibacterota. **Table S5.** The list of genes that are associated with the cellulose degradation to acetate and H_2_ production in the MAGs of Fibrobacterales. **Table S6.** The list of genes associated with protein degradation to acetate and H_2_ production in the MAGs of Bacteroidales. **Table S7.** The list of genes associated with oleic acid degradation to acetate and H_2_ production in the MAGs of Leptospiraceae. **Table S8.** The list of genes associated with protein and cellulose degradation to acetate and H_2_ production in the MAGs of Clostridiales. **Table S9.** The abundance of genes coding for the carbon monoxide dehydrogenase/acetyl-CoA synthase complex (*CODH/ACS*) in the metagenome data of the original sediment, control sample and treatments with different OMs at *t*_11_. **Table S10.** The list of genes that are associated with the “Wood–Ljungdahl” (WL) pathway in the MAGs of Desulfatiglandales. **Table S11.** The list of genes associated with methanogenesis in the MAGs of genus *Methanococcus*, genus *Methanocalculus* and order Methanosarcinales. **Fig.** **S1.** The changes in the cell number of uncultured microbes in response to the addition of different OMs. The cell number was calculated from the relative abundance and prokaryotic16S rRNA gene copy numbers. The prokaryotic16S rRNA gene copy numbers were shown in our previous study [1]; *t*_6_ and *t*_11_ indicate samples that were analyzed after 6 months and 11 months, respectively. **Fig.** **S2.** The comparison of prokaryotic communities at the phylum level in response to the addition of different OMs based on analysis of 16S rRNA gene amplicon; *t*_6_ and *t*_11_ indicate samples that were analyzed after 6 months and 11 months, respectively. **Fig.** **S3.** The comparison of prokaryotic communities at the RNA level in response to the addition of different OMs based on analysis of 16S rRNA amplicon. A: The relative abundance of *Methanococcus*, *Methanocalculus*, Methanosarcinales, *Ca. *Bathyarchaeota, *Ca. *Woesearchaeota, *Ca. *Fermentibacterota, Fibrobacterales, Bacteroidales, Fusibacter, Clostridiales, Syntrophotalea, Leptospiraceae and Dehalococcoidia. B: The prokaryotic communities at the phylum level. In ^12^C- DIC treatments, four samples collected at *t*_6_ and *t*_11_ of each substrate and control were mixed and used to RNA extraction. **Fig.** **S4.** The comparison of prokaryotic communities in response to the addition of different OMs based on analysis of metagenomic reads. A: The relative abundance of *Methanococcus*, *Methanocalculus*, Methanosarcinales, *Ca. *Bathyarchaeota, *Ca. *Woesearchaeota, *Ca. *Fermentibacterota, Fibrobacterales, Bacteroidales, Fusibacter, Clostridiales, Syntrophotalea, Leptospiraceae and Dehalococcoidia. B: The prokaryotic communities at the phylum level. In ^12^C- DIC treatments, two samples collected at *t*_11_ of each substrate and control were mixed and used to DNA extraction and metagenomic sequencing. **Fig.** **S5.** Abundance of genes involved in OC degradation in the metagenome data of original sediment and the addition of different OMs at *t*_11_.

## Data Availability

Metagenomic-assembled sequences and all MAGs from the current study have been deposited in eLMSG (an eLibrary of Microbial Systematics and Genomics, https://www.biosino.org/elmsg/index) under accession numbers LMSG_G000011456.1-LMSG_G000011484.1. Sequences of Illumina sequencing raw data and sequences were submitted to GenBank of NCBI under accession numbers PRJNA899565.

## Abbreviations

OC: Organic carbon
IC: Inorganic carbon
MAG: Metagenome assembly genome
MZ: Methanogenic zone
DOC: Dissolved organic carbon
hox NAD: Reducing hydrogenase
hnd NADP: Reducing hydrogenase
frhB: Coenzyme F420 hydrogenase subunit beta
WL: Wood–Ljungdahl
NCBI: National Center for Biotechnology Information
KEGG: Kyoto Encyclopedia of Genes and Genomes
GTDB: Genome Taxonomy Database
DIC: Dissolved inorganic carbon
LC-IRMS: Liquid chromatography coupled to isotope ratio mass spectrometry
*bcr*: Benzoyl-CoA reductase
*acss*: Acetyl-CoA synthetase
*pta-ack*: Phosphotransacetylase-acetate kinase
*aor*: Aldehydes
*por*: Pyruvate/2-ketoisovalerate
*ior*: Indolepyruvate
*vor*: Oxoisovalerate
*kor*: 2-Oxoglutarate
*lol*: Lipoprotein transporter
*cbh*: 1,4-Beta-cellobiosidase
*CODH/ACS*: Carbon monoxide dehydrogenase/acetyl-CoA synthase complex
DEPC: Diethylpyrocarbonate
SD: Standard deviation
DNA-SIP: DNA-based stable isotope probing
DNA/RNA-SIP: DNA/RNA-based stable isotope probing
