# Supplementary material for: Anaerobic degradation of organic carbon supports uncultured microbial populations in estuarine sediments
Source: Microbiome. 2023 Apr 20;11:81. doi: 10.1186/s40168-023-01531-z (PMC10116835; doi:10.1186/s40168-023-01531-z)
Supplement: Supplementary file 2 — Additional file 1: Table S1. The list of samples used for DNA/RNA isolation and acetate measurements. Table S2. The overview of MAGs that were analyzed in this study. Table S3. The list of genes that are associated with benzoate degradation to acetate and H2 production in the MAGs of Dehalococcoidia. Table S4. The list of genes that are associated with the cellulose degradation to acetate and H2 production in the MAGs of Ca. Fermentibacterota. Table S5. The list of genes that are associated with the cellulose degradation to acetate and H2 production in the MAGs of Fibrobacterales. Table S6. The list of genes associated with protein degradation to acetate and H2 production in the MAGs of Bacteroidales. Table S7. The list of genes associated with oleic acid degradation to acetate and H2 production in the MAGs of Leptospiraceae. Table S8. The list of genes associated with protein and cellulose degradation to acetate and H2 production in the MAGs of Clostridiales. Table S9. The abundance of genes coding for the carbon monoxide dehydrogenase/acetyl-CoA synthase complex (CODH/ACS) in the metagenome data of the original sediment, control sample and treatments with different OMs at t11. Table S10. The list of genes that are associated with the “Wood–Ljungdahl” (WL) pathway in the MAGs of Desulfatiglandales. Table S11. The list of genes associated with methanogenesis in the MAGs of genus Methanococcus, genus Methanocalculus and order Methanosarcinales. Fig. S1. The changes in the cell number of uncultured microbes in response to the addition of different OMs. The cell number was calculated from the relative abundance and prokaryotic16S rRNA gene copy numbers. The prokaryotic16S rRNA gene copy numbers were shown in our previous study [1]; t6 and t11 indicate samples that were analyzed after 6 months and 11 months, respectively. Fig. S2. The comparison of prokaryotic communities at the phylum level in response to the addition of different OMs based on analysis of 16S rRNA gene [file 40168_2023_1531_MOESM1_ESM.zip › Supplementary Information.docx]

Supplementary Information

**Anaerobic degradation of** **organic carbon supports uncultured microbial populations in** **estuarine sediments**

Tiantian Yu^1,2^, Weichao Wu^3,4,5^, Wenyue Liang^2^, Yinzhao Wang^2^, Jialin Hou^2^, Yunru Chen^2^, Marcus Elvert^3,4^, Kai-Uwe Hinrichs^3,4^, Fengping Wang^1,2,*^

^1^School of Oceanography, Shanghai Jiao Tong University, 200240 Shanghai, China;

^2^State Key Laboratory of Microbial Metabolism, School of Life Sciences and Biotechnology, Shanghai Jiao Tong University, 200240 Shanghai, China;

^3^Organic Geochemistry Group, MARUM-Center for Marine Environmental Sciences, University of Bremen, 28359 Bremen, Germany;

^4^Faculty of Geosciences, University of Bremen, 28359 Bremen, Germany;

^5^Shanghai Engineering Research Center of Hadal Science and Technology, College of Marine Science, Shanghai Ocean University, 201306 Shanghai, China.

* To whom correspondence should be addressed. E-mail: fengpingw@sjtu.edu.cn

All of our authors have agreed to submit the manuscript to the Microbiome journal. There is no conflict of interests from the authors.

**Supplementary Tables**

Table S1. The list of samples used for DNA/RNA isolation and acetate measurements.

| Sampling Scheme | Original sample | ^12^C-DIC | | | | ^13^C-DIC | | | |
| --- | --- | --- | --- | --- | --- | --- | --- | --- | --- |
|  |  | t_6_ | | t_11_ | | t_6_ | | t_11_ | |
|  |  | Repetition-1 | Repetition-2 | Repetition-1 | Repetition-2 | Repetition-1 | Repetition-2 | Repetition-1 | Repetition-2 |
| 16S rRNA gene quantification | √ | √ | √ | √ | √ |  |  |  |  |
| 16S rRNA gene diversity (DNA-level) | √ | √ | √ | √ | √ |  |  |  |  |
| 16S rRNA diversity (RNA-level) | √ | √ | | | |  |  |  |  |
| Metagenome | √ |  |  | √ | |  |  |  |  |
| Carbon isotopic composition and amount of acetate |  | √ | √ | √ | √ | √ | √ | √ | √ |

Table S2. The overview of MAGs that were analyzed in this study.

Table S3. The list of genes that are associated with benzoate degradation to acetate and H_2_ production in the MAGs of Dehalococcoidia.

Table S4. The list of genes that are associated with the cellulose degradation to acetate and H_2_ production in the MAGs of *Ca.* Fermentibacterota.

Table S5. The list of genes that are associated with the cellulose degradation to acetate and H_2_ production in the MAGs of Fibrobacterales.

Table S6. The list of genes associated with protein degradation to acetate and H_2_ production in the MAGs of Bacteroidales.

Table S7. The list of genes associated with oleic acid degradation to acetate and H_2_ production in the MAGs of Leptospiraceae.

Table S8. The list of genes associated with protein and cellulose degradation to acetate and H_2_ production in the MAGs of Clostridiales.

Table S9. The abundance of genes coding for the carbon monoxide dehydrogenase/acetyl-CoA synthase complex (*CODH/ACS*) in the metagenome data of the original sediment, control sample and treatments with different OMs at t_11_.

Table S10. The list of genes that are associated with the “Wood–Ljungdahl” (WL) pathway in the MAGs of Desulfatiglandales.

Table S11. The list of genes associated with methanogenesis in the MAGs of genus *Methanococcus*, genus *Methanocalculus* and order Methanosarcinales.

**Supplementary Figures**

**
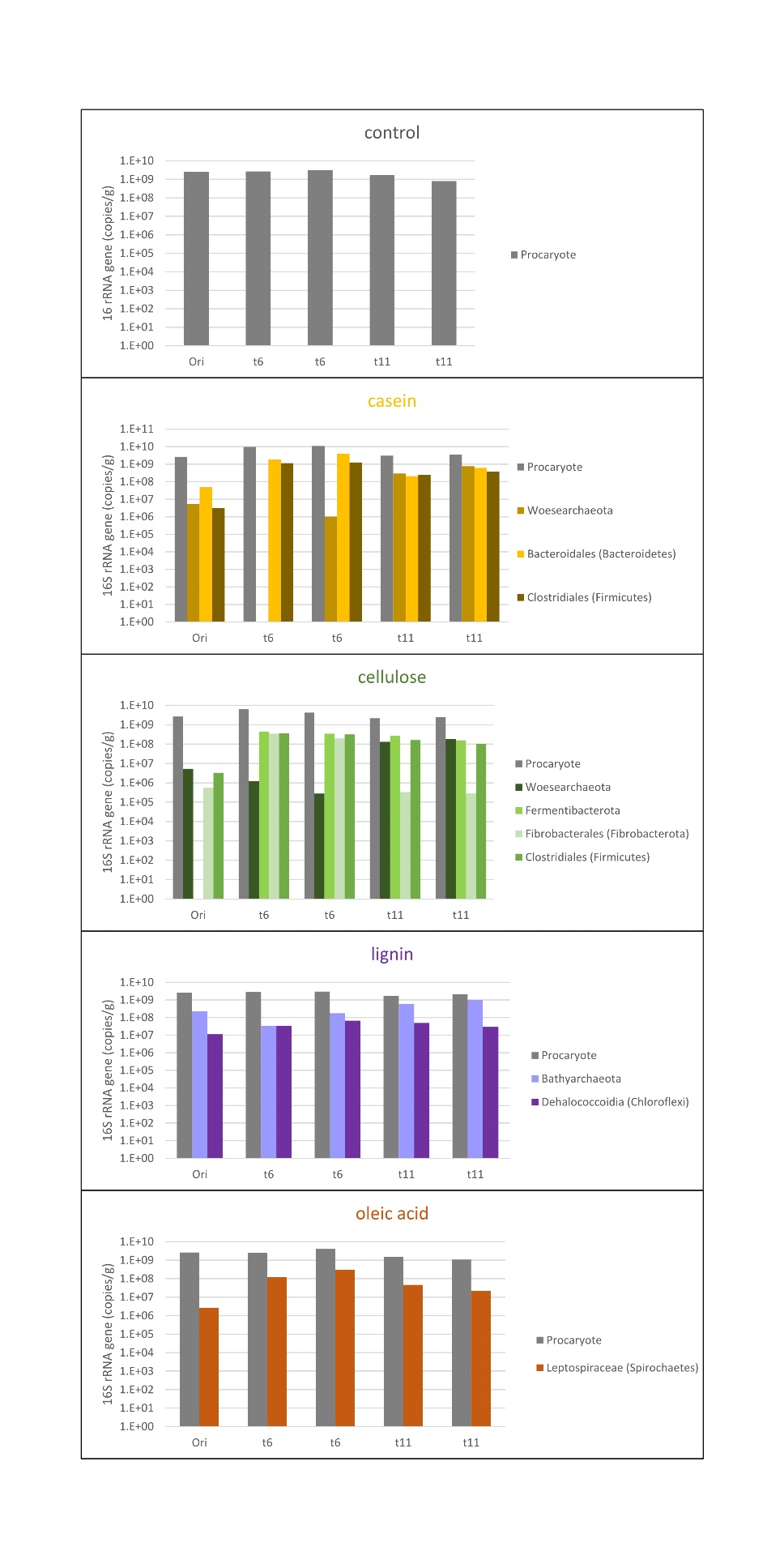
**

Fig. S1. The changes in the cell number of uncultured microbes in response to the addition of different OMs. The cell number was calculated from the relative abundance and prokaryotic16S rRNA gene copy numbers. The prokaryotic16S rRNA gene copy numbers were shown in our previous study [1]; t_6_ and t_11_ indicate samples that were analyzed after 6 months and 11 months, respectively.


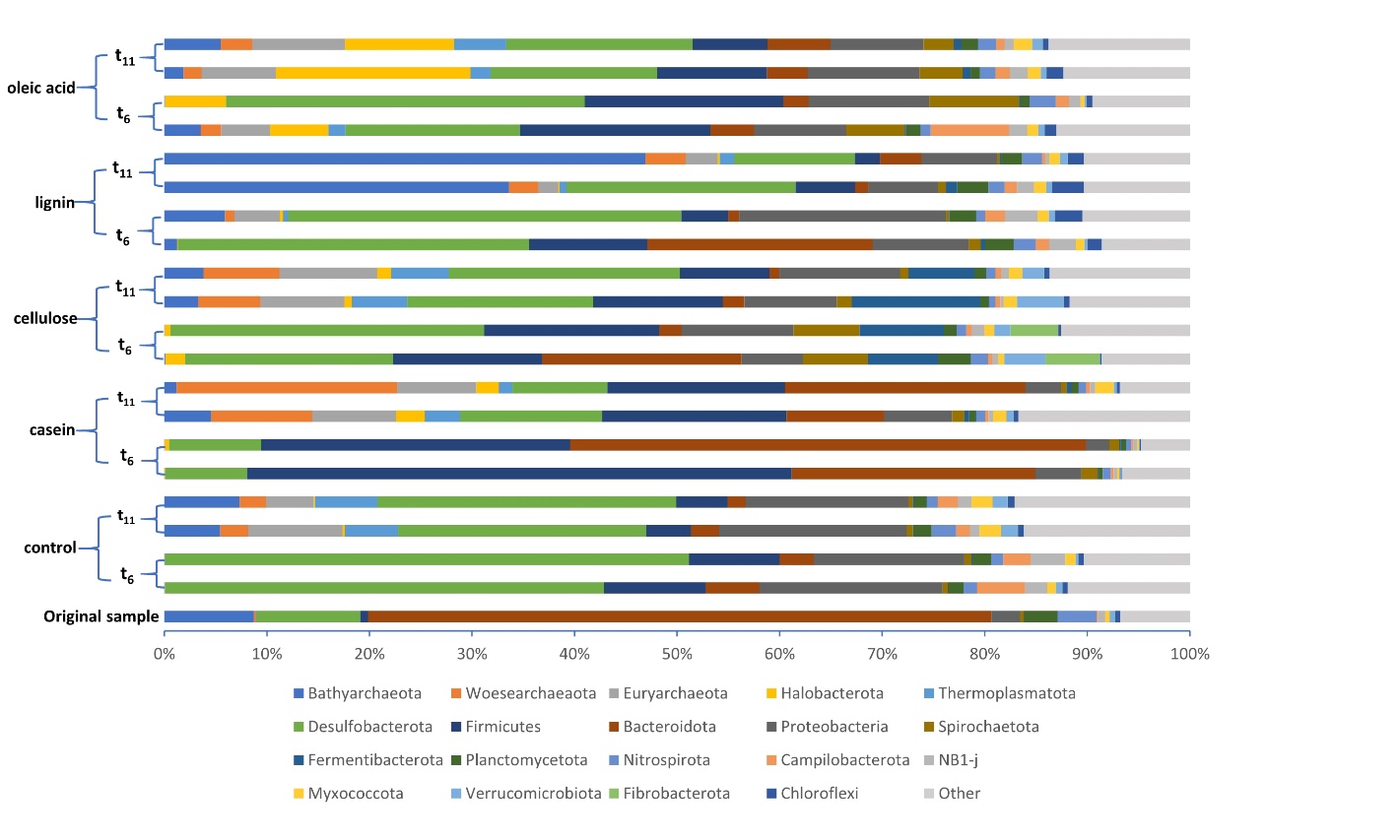


Fig. S2. The comparison of prokaryotic communities at the phylum level in response to the addition of different OMs based on analysis of 16S rRNA gene amplicon; t_6_ and t_11_ indicate samples that were analyzed after 6 months and 11 months, respectively.


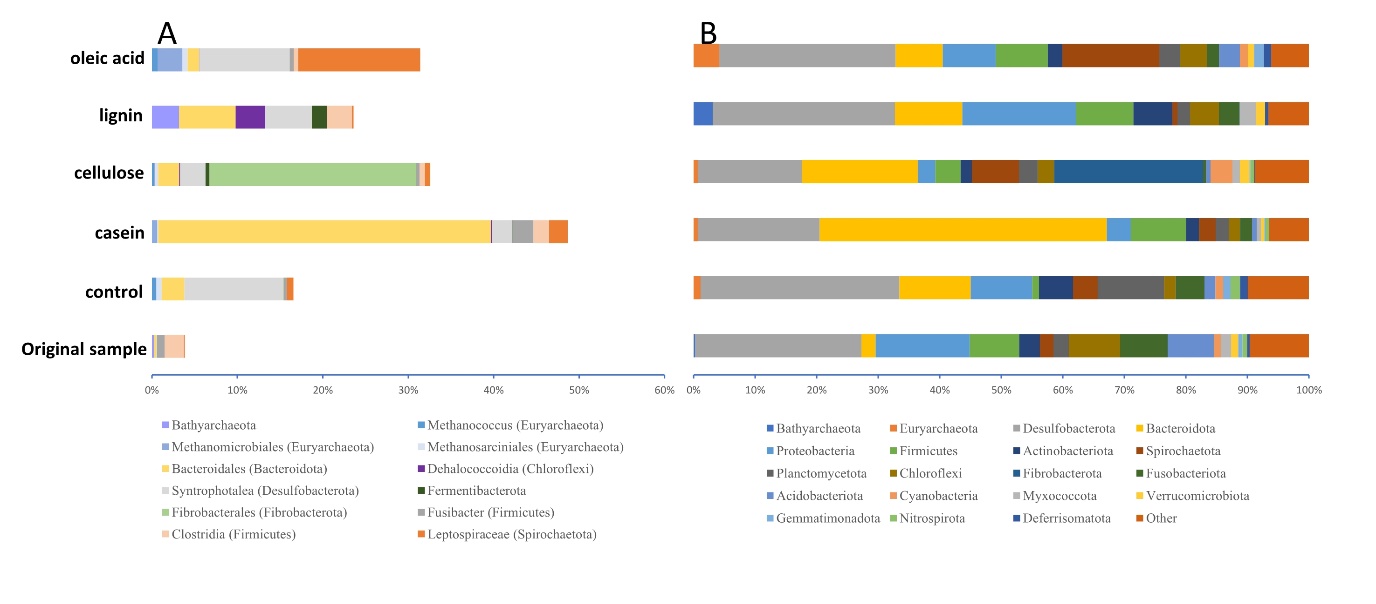


Fig. S3. The comparison of prokaryotic communities at the RNA level in response to the addition of different OMs based on analysis of 16S rRNA amplicon. A: The relative abundance of *Methanococcus*, *Methanocalculus*, Methanosarcinales, *Ca.* Bathyarchaeota, *Ca.* Woesearchaeota, *Ca.* Fermentibacterota, Fibrobacterales, Bacteroidales, Fusibacter, Clostridiales, Syntrophotalea, Leptospiraceae and Dehalococcoidia. B: The prokaryotic communities at the phylum level. In ^12^C- DIC treatments, four samples collected at t_6_ and t_11_ of each substrate and control were mixed and used to RNA extraction.


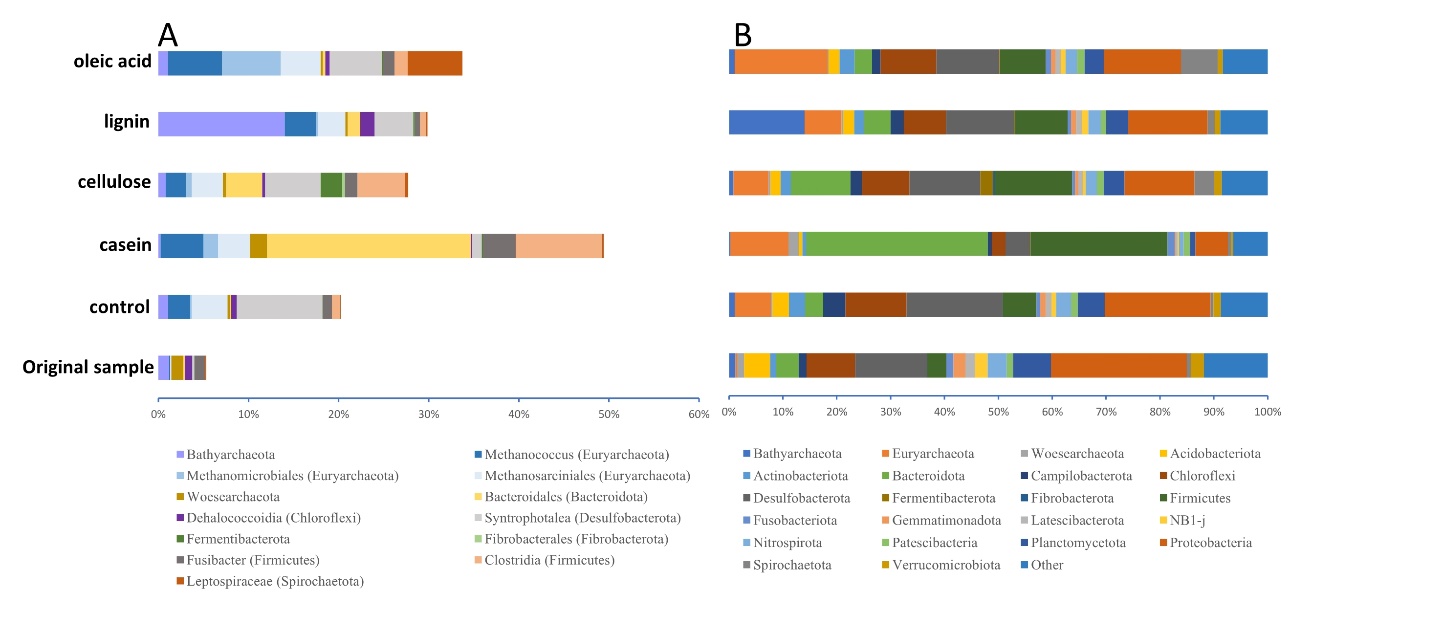


Fig. S4. The comparison of prokaryotic communities in response to the addition of different OMs based on analysis of metagenomic reads. A: The relative abundance of *Methanococcus*, *Methanocalculus*, Methanosarcinales, *Ca.* Bathyarchaeota, *Ca.* Woesearchaeota, *Ca.* Fermentibacterota, Fibrobacterales, Bacteroidales, Fusibacter, Clostridiales, Syntrophotalea, Leptospiraceae and Dehalococcoidia. B: The prokaryotic communities at the phylum level. In ^12^C- DIC treatments, two samples collected at t_11_ of each substrate and control were mixed and used to DNA extraction and metagenomic sequencing.


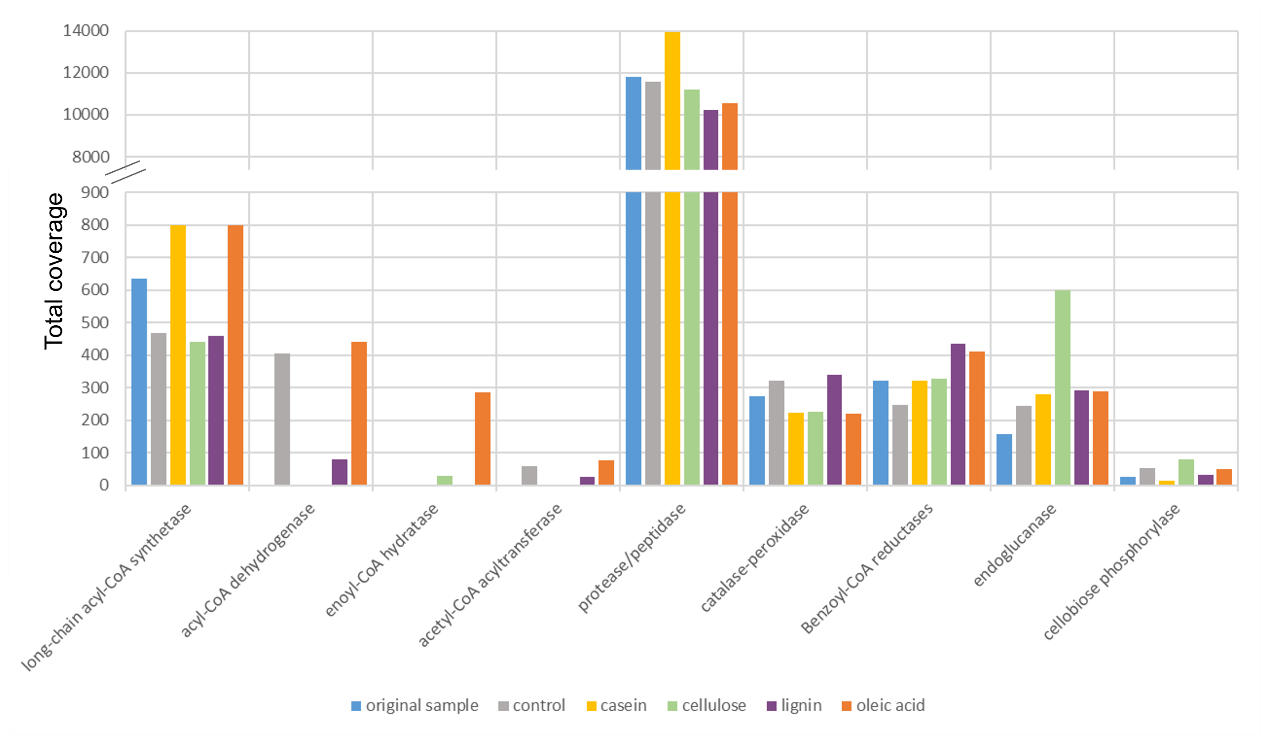


Fig. S5. Abundance of genes involved in OC degradation in the metagenome data of original sediment and the addition of different OMs at t_11_.

**Reference**

1. Yu T, Wu W, Liang W, Lever MA, Hinrichs K-U, Wang F. Growth of sedimentary Bathyarchaeota on lignin as an energy source. Proc Natl Acad Sci USA. 2018;115:6022-6027.
